# Ultrasound-targeted microbubble destruction enhances gene transduction of adeno-associated virus in a less-permissive cell type, NIH/3T3

**DOI:** 10.3892/mmr.2013.1560

**Published:** 2013-06-27

**Authors:** LIFANG JIN, FAN LI, HUIPING WANG, YUNHUA LI, FANG WEI, LIANFANG DU

**Affiliations:** 1 Department of Ultrasound, Shanghai First People's Hospital, Shanghai Jiao Tong University, Shanghai 200080, P.R. China; 2 Experimental Research Center, Shanghai First People's Hospital, Shanghai Jiao Tong University, Shanghai 200080, P.R. China

**Keywords:** ultrasonics, microbubbles, adeno-associated virus, induced pluripotent stem cells, sonoporation

## Abstract

Adeno-associated virus (AAV) is a common vector utilized in gene therapy. The NIH/3T3 cell line, which is a potential induced pluripotent stem (iPS) cell type, was identified to be a less-permissive cell type to AAV due to its defective endosomal processing. Ultrasound-targeted microbubble destruction (UTMD) enhanced the gene transduction of AAV in permissive cells. However, there are no data concerning UTMD enhancement in less-permissive cells, and the exact mechanism of UTMD enhancement in cellular uptake is unclear. Greater knowledge concerning the rate-limiting steps in NIH/3T3 cells would aid in the elucidation of the mechanism of UTMD enhancement in the gene transduction of AAV. In the present study, UTMD enhanced the gene transduction of AAV in NIH/3T3 cells, suggesting that UTMD-enhanced AAV-mediated gene transduction may be beneficial for gene therapy in iPS cells. The dose dependence of UTMD enhancement indicated that mechanisms other than sonoporation were involved in the cellular uptake of AAV. However, UTMD did not greatly increase the gene transduction of AAV in NIH/3T3 cells. Additionally, the similar degree of enhancement in the two cell types resulted in no correlation between UTMD and endosomal processing. Future studies on UTMD-mediated AAV transduction in other non- or less-permissive cell types may aid in elucidating the exact mechanism of UTMD enhancement in cellular uptake.

## Introduction

Adeno-associated virus (AAV) is a small, single-stranded DNA-containing, non-pathogenic, human parvovirus. AAV has been widely utilized as a vector for gene therapy in various cell and tissue types (1–5). However, a number of non- and less-permissive cell types have been identified (6,7) and these cell types were not infected efficiently by AAV.

Mouse fibroblast NIH/3T3 cells have been used to develop induced pluripotent stem (iPS) cells (8). The iPS cells may provide new opportunities for modeling human diseases and the potential for personalized regenerative cell therapies (9). However, gene therapy in NIH/3T3 cells that is mediated by the AAV vector is limited, as NIH/3T3 cells have been identified to be a less-permissive cell type (10,11).

Ultrasound-targeted microbubble destruction (UTMD) has efficiently and safely enhanced AAV-mediated gene transduction in certain permissive cell types (12–15). However, there are no studies concerning the increased AAV-mediated gene transduction with the application of UTMD in the less-permissive cell type, NIH/3T3.

The exact mechanism whereby UTMD enhances cellular uptake has not yet been elucidated. However, the theory of sonoporation is generally accepted (16–18), in which microbubbles exposed to ultrasound generate microstreams or microjets, which create shear stress on cells and open transient pores in cell membranes (19). These transient pores are suggested to facilitate the cellular uptake of extracellular material, and entry through such pores is proposed to be direct and rely on endocytosis (17). However, it has also been suggested that endocytosis is essential for UTMD enhancement in cellular uptake, which is not concordant with the theory of sonoporation (20–23).

NIH/3T3 cells were identified to be a less-permissive cell type due to their defective endosomal processing (10,11). It is assumed that if UTMD is able to greatly enhance the AAV-mediated gene transduction in the NIH/3T3 cells, this would suggest that UTMD influences the rate-limiting steps of NIH/3T3 cells. This is significant when investigating the correlation between UTMD and endosomal processing.

The present study applied UTMD as a technique to enhance the gene transduction of AAV in a less-permissive cell type, NIH/3T3. The UTMD parameters were optimized and the gene transduction enhancement, dose dependence and cell viability in NIH/3T3 cells was compared with a permissive cell type, HeLa. Studies of the two cell types would aid in determining the value of UTMD in AAV-mediated gene transduction, and in elucidating the mechanism of UTMD facilitation in cellular uptake.

## Materials and methods

### Cell culture

NIH/3T3 and HeLa cells were maintained in Dulbecco's modified Eagle's medium (DMEM; Gibco, Carlsbad, CA, USA) at 37°C and 5% CO_2_. The medium was supplemented with 10% fetal bovine serum (Gibco). The cells were seeded into alternative wells of 24-well plates, 24 h prior to infection. To achieve 90% confluency, 1×10^5^ HeLa cells and 5×10^4^ NIH/3T3 cells were seeded in each well.

### Virus infection

When the cells had been infected, the medium was replaced with 150 μl complete DMEM containing recombinant AAV serotype 2 (rAAV2) vector encoding the enhanced green fluorescent protein (EGFP) gene (rAAV2-EGFP; Beijing FivePlus Molecular Medicine Institute, Beijing, China). Fresh DMEM (350 μl) was added to the wells 2 h post-infection, and the medium containing the virus was replaced with 500 μl complete DMEM 24 h following treatment.

In the dose-effect experiments, the cells were infected with rAAV2-EGFP only. The doses of the virus were expressed as the multiplicity of infection (MOI). Six MOIs were investigated for each cell type following several preliminary tests. For HeLa cells, 0, 1,000, 2,000, 4,000 and 16,000 vector genome (v.g.)/cell were investigated. For NIH/3T3 cells, 0, 1,000, 10,000, 100,000 and 500,000 v.g./cell were investigated. In the remaining experiments, various MOIs were selected for respective purposes.

### UTMD protocols

A therapeutic ultrasound machine (Physioson-Basic; Physioson Elektromedizin AG, Laipersdorf, Germany) was used to emit ultrasound at a frequency of 1 MHz. The adjustable ultrasound parameters included the ultrasound intensity, exposure time and pulse output ratio. The ultrasound transducer was placed at the bottom of the 24-well plates with a small amount of coupling medium on the surface of the probe, which had an area of 2.5 cm^2^.

Microbubbles (Sonovue, Bracco, Milan, Italy) were lipid-shelled ultrasound contrast agents containing sulfur hexafluoride gas (diameter, 2.5–6.0 μm) and used at a concentration of ~2×10^8^ bubbles/ml. The volumetric ratio of microbubbles to medium dictated the dose of the contrast agent to be used.

To optimize the parameters, six combinations of ultrasound and microbubble parameters were investigated according to the results of a previous study (13). The parameters were combined according to the following sequence: Ultrasound intensity, exposure time, pulse output ratio and volume ratio of microbubbles.

### Gene transfection efficiency assays

At 48 h post-treatment, the EGFP transfection efficiency was evaluated by fluorescence microscopy and flow cytometry. Green fluorescence was detected using inverted fluorescence microscopy (Zeiss Axiovert S100; Carl Zeiss, Jena, Germany). The percentage of infected cells was measured by flow cytometry (FACSCalibur; BD Biosciences, Franklin Lakes, NJ, USA) following trypsinization and centrifugation. The treatment groups comprised AAV (treatment with rAAV2-EGFP alone) and UTMD+AAV (treatment with rAAV2-EGFP followed by UTMD). The measurements for each group were conducted in triplicate (one well per replicate).

### Cell viability assays

WST-8 was the effective constituent of the Cell Counting Kit-8 (CCK-8; Dojindo Molecular Technologies, Inc., Kumamoto, Japan) in the cell viability tests, as it is reduced to yellow water-soluble formazan by the dehydrogenase released from mitochondria. The quantity of formazan is in proportion to the number of living cells. Optical density (OD) values of the cell medium were detected to quantify the formazan levels and the corresponding cell viability.

The medium of the cells was changed to 500 μl fresh DMEM 2 h following infection and UTMD treatment. Immediately, the cells were added to 50 μl CCK-8 reagent, and then incubated for 2 h. Following this, 100 μl medium from each well of the 24-well plates was extracted and transferred to 96-well plates. The resulting color was analyzed using a microplate absorbance reader (iMark, Bio-Rad, Hercules, CA, USA) and the OD values were measured at 450 nm. Cells that were not infected or treated with UTMD served as the control group. The measurements for each group were conducted in quadruplicate (one well per replicate).

### Reverse transcription PCR (RT-PCR) and real-time PCR (qPCR) assay

Cells were trypsinized and harvested from the 24-well plates (six wells per group) 48 h following infection and UTMD treatment. The cells were lysed, and the total RNA was extracted from respective samples using TRIzol^®^ reagent (Invitrogen Life Technologies, Carlsbad, CA, USA), according to the manufacturer's instructions. The quantity and purity of the isolated RNA was measured by spectrophotometry. Reverse transcription to synthesize cDNA was conducted using the First Strand cDNA Synthesis kit (Promega Corporation, Madison, WI, USA). Quantitative PCR was performed on a 7500 Real-Time PCR System (Applied Biosystems, Inc., Foster City, CA, USA) using GoTaq^®^qPCR Master mix (Promega Corporation) and primers were designed against EGFP. The primer sequences used were: Forward: 5′-AGAAGAACGGCATCAAGGTG-3′ and reverse: 5′-GAACTCCAGCAGGACCATGT-3′. The conditions used for the reaction were: One cycle at 50°C for 2 min and 95°C for 10 min, and 40 cycles at 95°C for 15 sec and 60°C for 15 sec. Quantification, using the 2^−ΔΔCT^ analytical method, was performed in triplicate with β-actin as the internal standard.

### Western blot analysis

At 48 h following infection and UTMD treatment, cells were harvested from 24-well plates (24 wells per group). To detect the EGFP protein, the samples were separated on a sodium dodecyl sulfate-polyacrylamide gel (10% acrylamide) and blotted onto a nitrocellulose membrane. The membrane was then blocked with 0.2% I-Block (Sigma-Aldrich, St. Louis, MO, USA) in Tris-buffered saline supplemented with 0.1% Tween 20 (TBST) for 1 h at room temperature. Following incubation with goat polyclonal anti-EGFP antibody (1:1,000 in TBST ab111258; Abcam, Cambridge, UK) overnight at 4°C, the membrane was washed three times in TBST and incubated for 2 h with a peroxidase-conjugated anti-goat immunoglobulin G antibody (1:5,000 in TBST). The membrane was washed again, incubated for 1 min with SuperSignal West Pico Chemiluminescent Substrate (Pierce Biotechnology, Inc., Rockford, IL, USA) and exposed to Biomax Light Film (Kodak-Industrie, Chalon-sur-Saône, France).

### Statistical analysis

Data were expressed as mean ± standard deviation. One-way analysis of variance (ANOVA) with Bonferroni adjustment was used to determine the differences among groups in the UTMD parameter optimization and cell viability experiments. The independent samples t-test was used to detect differences between the treatment and control groups in the gene transduction efficiency detection and PCR experiments. P<0.05 was considered to indicate a statistically significant difference. Statistical analyses were performed using SPSS software (version 13.0; SPSS Inc., Chicago, IL, USA).

## Results

### UTMD parameter optimization

**Figure 1:**
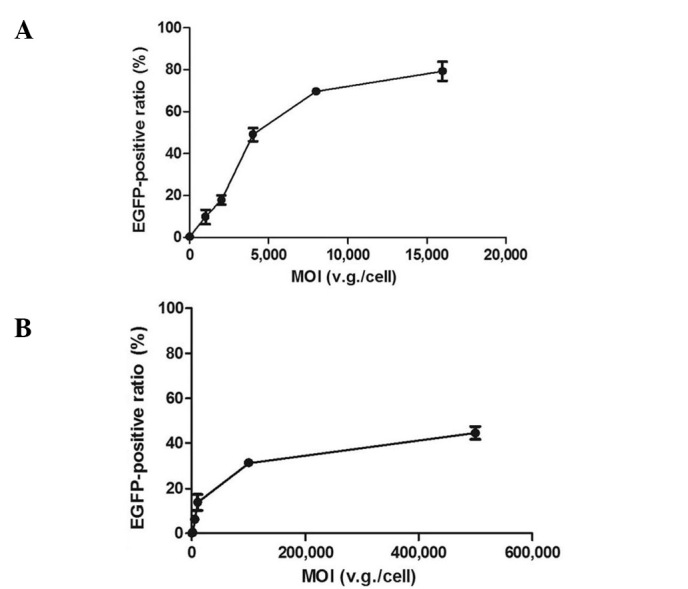
Dose-effect curves of the gene transduction of adeno-associated virus (AAV) in (A) HeLa and (B) NIH/3T3 cells. The cells were infected with recombinant AAV serotype 2 (rAAV2)-enhanced green fluorescent protein (EGFP) only. The EGFP-positive ratios were obtained by flow cytometry. There were three replicates in each group. MOI, multiplicity of infection; v.g., vector genome.

When the cells were infected with rAAV2-EGFP only, the transduction efficiency increased as the MOIs were elevated in both cell types. In the dose-effect curves, the effects markedly increased in the ascending curve segment and gradually in the plain segment (Fig. 1). To obtain the MOI in the later enhancement experiments, the approximate midpoint of the ascending segment was selected. To explore the dose dependence of UTMD enhancement, a lower and higher dose were also selected. Therefore, the MOIs of 1,000, 2,000 and 4,000 v.g./cell were selected for HeLa cells, and 1,000, 10,000 and 50,000 v.g./cell for NIH/3T3 cells. To confirm the UTMD enhancement of gene transcription and protein expression, the MOI of 2,000 v.g./cell was selected for HeLa cells and 10,000 v.g./cell for NIH/3T3 cells.

**Figure 2:**
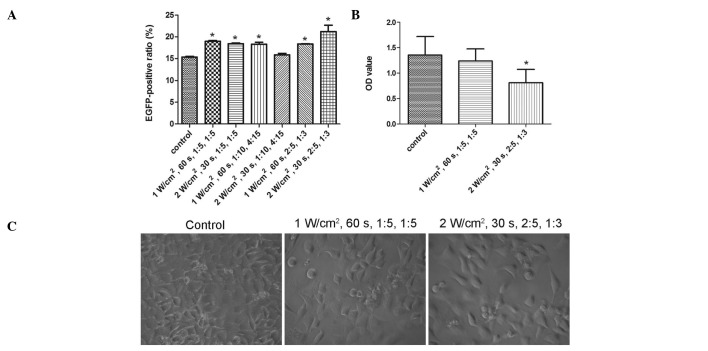
Ultrasound-targeted microbubble destruction (UTMD) parameter optimization in HeLa cells. (A) Comparison of the transduction efficiency among six UTMD parameter combinations. The enhanced green fluorescent protein (EGFP)-positive ratios were obtained from flow cytometry. The cells infected with recombinant adeno-associated virus serotype 2 (rAAV2)-EGFP alone served as the control group. The cells in the remaining groups were infected with rAAV2-EGFP and treated with different UTMD parameter combinations. ^*^P<0.05 vs. control. (B) Comparison of cell viability between two selected UTMD parameter combinations. The optical density (OD) values were obtained from the cell viability assays. Untreated cells served as the control group. The cells in the remaining groups were infected with rAAV2-EGFP and treated with two different UTMD parameter combinations. ^*^P<0.05 vs. control. (C) Light microscopy of HeLa cells in the same groups as (B). Original magnification, ×400.

All the combinations of UTMD parameters, with the exception of 2 W/cm^2^, 30 sec, 1:10, 4:15, significantly enhanced the transduction efficiency of rAAV2-EGFP (P=0.000, P=0.001, P=0.001, P=1.000, P=0.001 and P=0.000, from the first to the last combination, respectively) (Fig. 2A). The combination of 2 W/cm^2^, 30 sec, 2:5, 1:3 demonstrated the greatest increase in transduction efficiency, while that of 1 W/cm^2^, 60 sec, 1:5, 1:5 demonstrated the second greatest increase. However, due to the extreme parameters, such as 2 W/cm^2^ and 2:5, in the former combination, the two combinations were investigated in the cell viability tests.

A comparison of treated and untreated cells in the CCK-8 cell viability assays showed the combination of 2 W/cm^2^, 30 sec, 2:5, 1:3 to be harmful (P=0.007), whereas that of 1 W/cm^2^, 60 sec, 1:5, 1:5 was shown to be safe (P=1.000) (Fig. 2B). Results of the light microscopy examinations demonstrated that fewer cells remained after being treated with 2 W/cm^2^, 30 sec, 2:5, 1:3 compared with 1 W/cm^2^, 60 sec, 1:5, 1:5 (Fig. 2C). Therefore, the optimized UTMD parameter combination was defined as 1 W/cm^2^, 60 sec, 1:5, 1:5.

### Enhancement and dose dependence

**Figure 3:**
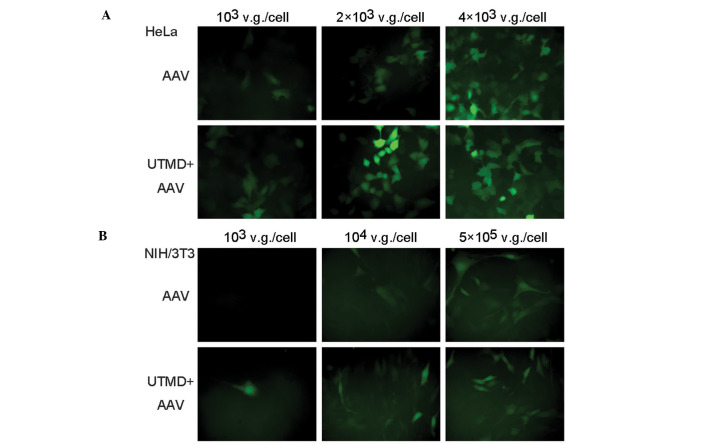
Fluorescence microscopy of enhanced green fluorescent protein (EGFP) expression. (A) Fluorescence microscopy of HeLa cells 48 h following infection with recombinant adeno-associated virus serotype 2 (rAAV2)-EGFP with three different multiplicities of infection (MOIs). The cells in the AAV group were infected with rAAV2-EGFP alone. The cells in the ultrasound-targeted microbubble destruction (UTMD)+AAV group were infected with rAAV2-EGFP and treated with optimized UTMD. (B) Fluorescence microscopy of NIH/3T3 cells 48 h following infection with rAAV2-EGFP with three different MOIs. v.g., vector genome.

In the fluorescence microscopic images, the number of green fluorescent HeLa cells in the UTMD+AAV group was greater than that in the AAV group when low and medium AAV doses were applied. The fluorescence intensity of the cells in the UTMD+AAV group was stronger than that in the AAV group. In addition, the UTMD enhancement was not as obvious when low and medium AAV doses were used as opposed to a high AAV dose (Fig. 3A). Similar results were observed in the NIH/3T3 cells (Fig. 3B).

**Figure 4:**
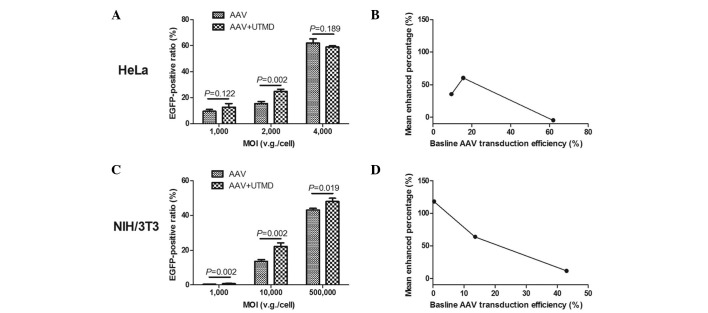
Dose dependence of ultrasound-targeted microbubble destruction (UTMD) enhancement in adeno-associated virus (AAV) transduction. (A) The enhanced green fluorescent protein (EGFP)-positive ratios of infected HeLa cells 48 h following infection of rAAV2-EGFP with three different multiplicities of infection (MOIs). The data were obtained using flow cytometry conducted in triplicates. (B) The baseline efficiency dependence of the mean enhanced transduction efficiency of HeLa cells. (C) The EGFP-positive ratios of infected NIH/3T3 cells 48 h following infection with rAAV2-EGFP with three different MOIs. (D) The baseline efficiency dependence of the mean enhanced transduction efficiency of NIH/3T3 cells. v.g., vector genome.

Data from the flow cytometry investigation demonstrated that UTMD enhancement was greatest in the HeLa and NIH/3T3 cells when medium doses of AAV were applied. The mean ratio of green fluorescent HeLa cells in the UTMD+AAV group was 24.96±1.42% at a MOI of 2,000 v.g./cell, which was significantly higher than that in the AAV group (15.56±1.64%) (P=0.002). In the HeLa cells, the absolute mean enhanced ratio was 9.4% and the UTMD enhanced transfection by 1.60-fold (Fig. 4A). The mean ratio of green fluorescent NIH/3T3 cells in the UTMD+AAV group was 22.28±1.89% at a MOI of 10,000 v.g./cell, which was also significantly higher than that in the AAV group (13.59±1.05%) (P=0.002). The absolute mean enhanced ratio was 8.69% and the UTMD enhanced transfection by 1.64-fold in the NIH/3T3 cells (Fig. 4C). As the baseline AAV transduction efficiency increased, the mean enhanced percentage of transduction efficiency initially increased and then decreased in the HeLa cells (Fig. 4B), but continued decreasing in the NIH/3T3 cells (Fig. 4D).

### Enhanced gene transcription and expression

**Figure 5:**
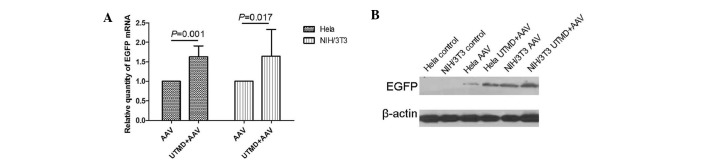
Ultrasound-targeted microbubble destruction (UTMD) enhancement in enhanced green fluorescent protein (EGFP) gene transcription and expression. (A) The relative quantity of EGFP mRNA detected by real-time PCR (qPCR) in HeLa and NIH/3T3 cells 48 h following infection with rAAV2-EGFP. (B) The relative quantity of EGFP protein detected by western blot analysis in HeLa and NIH/3T3 cells 48 h following infection with rAAV2-EGFP.

The PCR results revealed that, the relative quantity of EGFP gene transcription in the UTMD+AAV group was significantly greater than that in the AAV group in HeLa and NIH/3T3 cells (P=0.001 and P=0.017, respectively). The mean UTMD enhancement was 1.62±0.11-fold in the HeLa cells and 1.64±0.28-fold in the NIH/3T3 cells (Fig. 5A). The western blot analysis demonstrated that the relative quantity of EGFP gene expression in the UTMD+AAV group was greater than that in the AAV group in HeLa and NIH/3T3 cells (Fig. 5B).

### Cell viability

**Figure 6:**
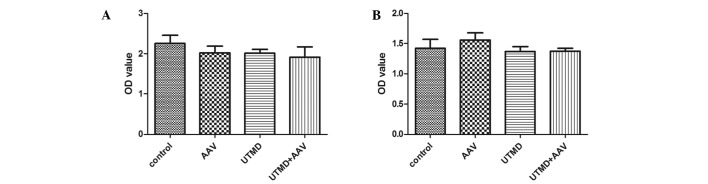
Effect of ultrasound-targeted microbubble destruction (UTMD) on cell viability. The cells in the UTMD group were treated with optimized UTMD only. Optical density (OD) values were obtained from the CCK-8 tests. The relative cell viability of (A) HeLa cells and (B) NIH/3T3 cells 2 h following treatment is shown.

The cell viability assays indicated that AAV infection, UTMD treatment and AAV infection with UTMD treatment did not significantly affect proliferation in the HeLa cells (P=0.640, P=0.558 and P=0.150, respectively) (Fig. 6A). Similarly, there was no significant difference in cell viability among the AAV, UTMD and UTMD+AAV groups (P=0.610, P=1.000 and P=1.000, respectively) in the NIH/3T3 cells (Fig. 6B).

## Discussion

In the present study, UTMD efficiently and safely enhanced the gene transduction of AAV in the less-permissive cell type, NIH/3T3. The enhancement effect of UTMD was 1.6 fold with regard to transduction efficiency and gene transcription. The results were similar to those of a previous study that demonstrated a 1.75-fold enhancement effect in another permissive cell type, retinal pigment epithelium (13). Furthermore, when UTMD along with corresponding parameters was applied *in vivo*, the enhancement effects were higher and sustained (13,15). Therefore, applying UTMD-mediated AAV transduction in *in vivo* studies of iPS cells in the gene therapy of inherited and acquired disorders may be beneficial (8,24,25).

The dose dependence of UTMD enhancement in the two cell types demonstrated inefficiency at high AAV doses. The inefficiency suggested saturation of the mechanism. The generally accepted mechanism of sonoporation is considered to facilitate direct entry of extracellular material through transit pores (17), and this route of entry would not become saturated as the extracellular material increased. Thus, sonoporation is not able to explain the dose dependence of the UTMD-enhanced AAV-mediated transduction. Other mechanisms may therefore be involved in the UTMD enhancement.

When using UTMD, the transduction efficiency was enhanced by 1.64-fold in the NIH/3T3 cells, and by 1.60 fold in the HeLa cells. Similarly, the enhanced gene transcription in the NIH/3T3 cells (1.64-fold) was also slightly greater than that in the HeLa cells (1.62-fold). Therefore, UTMD did not greatly enhance the gene transduction of AAV in the endosomal processing-defective cell type, NIH/3T3. In previous studies on NIH/3T3 cells, the enhanced gene transduction of AAV suggested an effect on the rate-limiting steps (10,26). However, the present study did not indicate that UTMD could bypass the defect in AAV trafficking in NIH/3T3 cells. The results were not able to identify a correlation between the mechanism of UTMD enhancement in cellular uptake and endosomal processing. Additional studies in other non- or less-permissive cell types with different AAV trafficking defects (27–30) are required to elucidate the exact mechanism of UTMD enhancement in AAV cellular uptake.

The parameters of UTMD used in the present study were taken from various studies (12,13,15,31). Therefore, the application of the UTMD parameters was determined by cell type, to a certain degree. The HeLa cell type was utilized to optimize the parameters. This was due to only one UTMD parameter combination being used in the enhancement in two cell types, in favor of comparison. HeLa cells were more tolerant than NIH/3T3 cells in the optimization experiment, and the optimized UTMD parameter combination significantly enhanced the gene transduction of AAV. Although enhancement was achieved utilizing optimized parameters for UTMD, there may be a greater increase in enhancement with the implementation of other methods, such as multiple and repeating operations of UTMD (32).

Overall, UTMD enhanced the gene transduction of AAV in the less-permissive cell type, NIH/3T3. UTMD-enhanced AAV-mediated gene transduction may be beneficial in iPS cell application. The degree of UTMD enhancement and the mode of dose dependence in NIH/3T3 cells were similar to those of the permissive cell type, HeLa. UTMD did not bypass the rate-limiting steps of AAV cellular trafficking in NIH/3T3 cells. Additional studies are required to elucidate the mechanism of UTMD enhancement and thus enable an increased enhancement.

## Abbreviations

AAV: adeno-associated virus
iPS: induced pluripotent stem
UTMD: ultrasound-targeted microbubble destruction
MOI: multiplicity of infection
CCK-8: cell counting kit-8
OD: optical density
